# Lipoprotein(a) Lowering—From Lipoprotein Apheresis to Antisense Oligonucleotide Approach

**DOI:** 10.3390/jcm9072103

**Published:** 2020-07-03

**Authors:** Maria Francesca Greco, Cesare R. Sirtori, Alberto Corsini, Marat Ezhov, Tiziana Sampietro, Massimiliano Ruscica

**Affiliations:** 1 Dipartimento di Science Farmacologiche e Biomolecolari, Università degli Studi di Milano, 20133 Milan, Italy; mariafrancesca.greco@unimi.it (M.F.G.); alberto.corsini@unimi.it (A.C.); 2 Dyslipidemia Center, A.S.S.T. Grande Ospedale Metropolitano Niguarda, 20162 Milan, Italy; cesare.sirtori@icloud.com; 3 IRCCS Multimedica, 20099 Milan, Italy; 4 National Medical Research Center of Cardiology of the Ministry of Health, Moscow, Russia; marat_ezhov@mail.ru; 5 U.O. Lipoapheresis and Center for Inherited Dyslipidemias, Fondazione Toscana Gabriele Monasterio, 56126 Pisa, Italy; tiziana.sampietro@ftgm.it

**Keywords:** lipoprotein(a), lipoprotein apheresis, statins, niacin, proprotein convertase subtilisin/kexin type 9, antisense oligonucleotide APO(a)L_rx_

## Abstract

It is well-known that elevated lipoprotein(a)—Lp(a)—levels are associated with a higher risk of cardiovascular (CV) mortality and all-cause mortality, although a standard pharmacotherapeutic approach is still undefined for patients with high CV risk dependent on hyperlipoproteinemia(a). Combined with high Lp(a) levels, familial hypercholesterolemia (FH) leads to a greater CVD risk. In suspected FH patients, the proportion of cases explained by a rise of Lp(a) levels ranges between 5% and 20%. In the absence of a specific pharmacological approach able to lower Lp(a) to the extent required to achieve CV benefits, the most effective strategy today is lipoprotein apheresis (LA). Although limited, a clear effect on Lp(a) is exerted by PCSK9 antagonists, with apparently different mechanisms when given with statins (raised catabolism) or as monotherapy (reduced production). In the era of RNA-based therapies, a new dawn is represented by the use of antisense oligonucleotides APO(a)L_rx_, able to reduce Lp(a) from 35% to over 80%, with generally modest injection site reactions. The improved knowledge of Lp(a) atherogenicity and possible prevention will be of benefit for patients with residual CV risk remaining after the most effective available lipid-lowering agents.

## 1. Introduction—An Overview

In the last decade, an awareness of the association between cardiovascular (CV) risk and elevated levels of lipoprotein(a) [Lp(a)] has emerged from both epidemiological and genetic studies [1,2,3] and led to the need to improve treatments for patients with atherosclerotic cardiovascular disease (ASCVD), as therapeutic approaches able to lower Lp(a) levels to the extent required to achieve a CV benefit are missing. Lipoprotein apheresis (LA) is still a potentially valuable therapeutic approach in the case of hyperlipoproteinemia(a) [4]. Lp(a) is the product of the covalent binding by the disulfide bond of apolipoprotein (apo)B (largely hydrophobic) to the plasminogen-like glycoprotein apo(a) (largely hydrophilic) in a 1:1 molar ratio [5,6]. Both apoB and apo(a) represent 80% of Lp(a) proteome, with 35 additional proteins that play roles from lipid metabolism to wound-healing, to immunity [7].

Clinical conditions resulting in raised CV risk are particularly stimulating, in order to improve choices for handling Lp(a) elevations. Familial hypercholesterolemia (FH) and elevated Lp(a) are a double heritable risk [8], i.e., carriers of a receptor-negative mutation in the low-density lipoprotein receptor (LDLR) gene with high Lp(a) (> 50 mg/dL) show a higher cardiovascular disease (CVD) risk compared to patients with the same mutation and Lp(a) levels < 50 mg/dL [9]. In suspected FH patients, the proportion of cases explained by biochemical evaluation of Lp(a) is between 5% and 20%. Genetically driven high levels of Lp(a) can raise LDL-cholesterol (LDL-C) and, independently, the risk of CVD [10]. Elevated levels of Lp(a) have been observed in FH patients, i.e., two- to three-fold, compared with controls matched for kringle IV 2 (KIV-2) copy number variation. Data from 46,200 Danish individuals showed that 25% of FH receive this diagnosis due to their high Lp(a) concentrations [11]. However, the mechanism behind the elevation of Lp(a) in FH is still unclear. Considering the hypothesis that Lp(a) may be catabolized by the low-density lipoprotein receptor (LDLR) pathway, Lp(a) levels rise with the number of LDLR mutations, consistent with a positive gene dosage effect [12]. However, the hypothesis of Lp(a) clearance through the LDLR has been challenged recently. No differences in Lp(a) levels were found between carriers of pathogenic variants of *LDLR* and *APOB* vs. noncarriers [13].

The uncertainties regarding the strategy to handle elevated Lp(a), particularly in patients with concomitant hypercholesterolemia, have paved the way to review the present-day evidence on the role of elevated Lp(a) in this high-risk condition, as well as on the relative value of different therapeutic approaches, i.e., from LA to upcoming RNA-based therapeutics.

Lp(a) levels are controlled mostly genetically by the *LPA* gene locus, characterized by an extensive size polymorphism of apo(a) caused by a variable number of different KIV-2 repeats. The size of Lp(a) is very broad, ranging from 300 to 800 kDa. The extensive polymorphic nature of the apo(a) gene size makes homozygotes of apo(a) size rare, whereas heterozygosity is at approximately 94% among Caucasians [14]. Individuals with small apo(a) isoforms have higher median levels of Lp(a) compared to individuals carrying large apo(a) isoforms. However, KIV-2 copy number variations alone explain 19–77% of the variation in Lp(a) levels [15]. Levels of Lp(a) vary among individuals of different ethnicities, with the African ethnicity having higher levels compared to the European and Asian ethnicities [16]. These observations are to be combined with the difficulties in establishing a generally accepted mode of measurement [17]. First of all, available assays report results in mass (mg/dL) instead of concentration in nmol/L, and a direct conversion may not be possible because of the variable number of repeated units in different apo(a) isoforms. Secondly, absolute differences in Lp(a) measurements for single samples have been reported as up to almost 80 mg/dL [18]. Third, Lp(a) values vary if samples used are fresh or have been frozen for prolonged periods of time. 

### Cardiovascular (CV) Risk Associated with Lipoprotein (a) Levels

The CV risk of elevated Lp(a) still remains an open question [19], in particular because of the unsettled definition of levels requiring treatment. Among the general population, Lp(a) concentrations have a more than 1000-fold interindividual range”, with a prevalence of 35% for values > 30 mg/dL, 20% for values > 60 mg/dL, 10% for values > 90 mg/dL, 5% for values > 116 mg/dL and 1% for values >180 mg/dL [20]. Data from a Mendelian randomization analysis indicated that the Lp(a)-lowering therapeutic effect size [to reduce CAD risk in a similar way to a reduction of 38.7 mg/dL (1 mmol/L)] of LDL-C should be roughly 100 mg/dL (2.6 nmol/L) [21]. This conclusion was not supported by other authors who replicated a similar approach, not considering, however, Lp(a)-elevating single-nucleotide polymorphisms (SNPs), but based on 13,781 individuals with median Lp(a) levels in a range typical of Caucasian populations. By this approach, it was calculated that an Lp(a) lowering of 65.7 mg/dL would be required in order to achieve a similar CV benefit as that attained by the 38.7 mg/dL reduction of LDL-C [22]. The above quoted value of 101.5 mg/dL [21] was likely overestimated, being based on patients with median Lp(a) concentrations two-fold to three-fold higher than the median levels found in the same ethnicity [23]. However, as elsewhere described, it cannot be overlooked that the analysis by Burgess et al. [21] included studies in which Lp(a) levels were lower than those defined as a treatment criterion for LA [24]. Finally, in the debate regarding whether or not high Lp(a) leads to recurrent CVD events, a population study concluded that in secondary prevention, an Lp(a) lowering by 50 mg/dL (approximately 105 nmol/L) over a period of 5 years is needed to reduce CVD events by 20% [25].

While an optimal Lp(a) level < 50 mg/dL was previously reported in Europe [26], the 2018 American Guidelines suggested the measurement of Lp(a) in people with a familial or personal history of premature atherosclerotic CVD that were not explained by major risk factors [27]. The 2019 European Society of Cardiology (ESC)/European Atherosclerosis Society (EAS) Guidelines for the management of dyslipidemias have recommended Lp(a) measurement at least once in each adult person’s lifetime, with the objective of identifying those with very high inherited Lp(a) levels (> 180 mg/dL) [28]. Nowadays, since Lp(a) levels remain stable throughout one’s life outside of acute illness, it seems there is not much role for serial measurement (cited in [17]). However, the value of 180 mg/dL has not been indicated as a threshold for Lp(a) risk, but rather as conveying a CVD-risk comparable to that of heterozygous familial hypercholesterolemia (HeFH). Thus, although the value of > 50 mg/dL—representing the 80th percentile of the Caucasian population—has been proposed as a tentative universal cut-off point [17], it remains a topic of debate among many experts in the lipid community [20]; while Lp(a) ≥ 50 mg/dL best predicted cardiovascular heart disease in the Caucasians, the Chinese-Americans and the Hispanics, the corresponding value for those of African ethnicity was 30 mg/dL [29]. A further challenge on this issue came from the genetic evidence linking high Lp(a) levels to the development of CVD, i.e., a stepwise increment in the Hazard Ratio (HR; from 1 to 2.6) was reported, starting between Lp(a) < 5 mg/dL and Lp(a) > 117 mg/dL [2].

In the case of FH patients carrying high levels of Lp(a), the mechanism beneath the increased risk may also act in the additive risk of the inhibition of fibrinolysis [30], as suggested by the seminal idea by Brown and Goldstein [31]. This and other hypotheses have never been proven, but have led to a number of contrasting studies, with some authors indicating a very high CV risk, and others not [32,33]. The measurement of “LDL-cholesterol” contains the cholesterol content of both LDL and the LDL component of Lp(a) [11], since each Lp(a) particle is composed of about 30−45% cholesterol. Interestingly, for a similar cholesterol content increase of Lp(a) and LDL (15 mg/dL; 0.39 mmol/L), Lp(a) cholesterol is more strongly associated with CV mortality; HR 1.18 (1.12–1.25) vs. 1.05 (1.04–1.07), respectively. A similar conclusion was reached in the case of all-cause mortality: HR 1.07 (1.04–1.10) for Lp(a) cholesterol and HR 1.01 (1.00–1.01) for LDL-C [34]. This evidence implies that both cholesterol content and the atherogenic nature of the apolipoprotein can be responsible for the increased risk [34,35]. Adjusting LDL-C concentration for Lp(a)-C improves the diagnostic accuracy of the Dutch Lipid Clinic Network and Simon Broome criteria, especially for patients with Lp(a) > 100 mg/dL and LDL-C < 251 mg/dL [36]. All-in-all, this evidence leads to the general suggestion of a priority screening of all FH patients for high Lp(a) [37]

The importance of dosing Lp(a) in FH patients was corroborated by Langsted et al. [11], reporting that the HR for myocardial infarction (MI), which is 1 for the individuals who are unlikely to have FH and Lp(a) < 50 mg/dL, rises to 1.4 in individuals who are likely to have FH and Lp(a) > 50 mg/dL, then to 3.2 in those with possible, probable or definite FH, and Lp(a) < 50 mg/dL, and finally to 5.3 in those with possibe or definite FH and Lp(a) > 50 mg/dL. More recently, in a large series of patients entering a Coronary Care Unit, evaluated using the same criteria, hyperlipoproteinemia(a) and FH were identified in 27% and 11.6% of individuals (vs. 4.4% of carriers of both disorders). Specifically, the risk of premature coronary artery disease was increased 1.9-fold when Lp(a) was considered, 3.2-fold in the case of FH phenotype, and 5.3-fold when both disorders were evaluated in combination [38]. An Italian and Swedish series showed that elevated Lp(a) significantly contributed to the raised CV risk in patients with a genomic diagnosis of FH [39]. It seems that FH does not cause hyperlipoproteinemia(a), but that a rise in Lp(a) increases the probability that an individual with genetic FH will be clinically recognized [13].

Although not in the scope of this review article, in addition to coronary artery disease [40], a raised incidence of aortic valve stenosis and coronary artery calcification has also been described in association with elevated Lp(a) [41,42,43,44]. As reviewed elsewhere [45,46], Lp(a) can exacerbate pathophysiological processes in calcific aortic disease, a progressive disorder that impairs the valve motion and restricts the ventricular outflow. Evidence also supports a causal association between Lp(a) and valve calcification.

## 2. Statins

Statins do not generally lower Lp(a) and may potentially raise Lp(a), although this trend seems confined to patients with a low molecular weight apo(a) phenotype [47]. A meta-analysis [48] involving 5256 patients (1271 on placebo and 3885 on different statins) from six randomized trials showed a difference in the statin vs. placebo pooled analysis, expressed as a geometric mean ratio of 1.11 (1.07–1.14). The mean changes from baseline ranged from +11.6% to +20.4% in the pravastatin group, and from +18.7% to +24.2% in the atorvastatin group. The authors also tested the effects of incubating HepG2 hepatocytes with atorvastatin, and reported a dose- and time-dependent increased expression of LPA mRNA, and synthesis and secretion of apo(a) protein [48]. Pirillo and Catapano [49] suggested that the underlying mechanism may relate to the reduction of intracellular cholesterol on the liver X receptor–farnesoid X receptor (LXR–FXR) axis, since an FXR-responsive element is located within the promoter of the LPA gene [50]. In a follow-up comment, Banach and Penson [51] noted that most of the listed trials had mean Lp(a) < 30 mg/dL, and thus, although some of the reported rises were up to 101 mg/dL, in most cases these changes may not have been clinically relevant. Specifically, in subjects with Lp(a) ≥ 30–50 mg/dL in the statin group, the mean absolute Lp(a) change was 18.1 mg/dL, whereas in those with Lp(a) ≥ 50 mg/dL, the mean absolute change was 10.3 mg/dL [52]. 

Another mechanism has been hypothesized by Ma et al. [53]. In statin-treated patients, the fractional turnovers of apo(a) and apoB proteins within Lp(a) are similar, thus reflecting a tight coupling of these protein components. A recent kinetic study relative to the production rate of Lp(a) suggested that the hepatic availability of apoB is unlikely to be rate-limiting for the assembly and production of Lp(a) particles [54]. Overall, the molecular mechanisms responsible for Lp(a) catabolism still remain elusive, with several receptor systems being proposed as implicated in this process (lipoprotein receptors, toll-like and scavenger receptors, lectins, and plasminogen receptors) [55].

In a recent individual-patient meta-analysis of 29,069 patients from seven randomized controlled trials (RCTs) with statins, both baseline and on-statin Lp(a) concentrations were associated with an almost linear association with increment in CVDs, particularly for patients with Lp(a) concentrations of 50 mg/dL or higher, and those on statin therapy [56]. Thus, it is reasonable to use caution in the use of statins in patients with significant Lp(a) elevations, although the LDL-C lowering effect will most likely prevail in terms of CV prevention (Table 1).

## 3. Nicotinic Acid

This somewhat obsolete lipid-lowering agent was the first one to provide convincing data on a potential Lp(a) lowering effect. Prominent in this area was the study by Chennamsetty et al., demonstrating that feeding transgenic-APOA mice 1% nicotinic acid (NA) reduced plasma APOA and hepatic expression of APOA [64]. Moving to humans, the Swedish group headed by Carlson indicated a decrease of 38% (95% CI 28–47%) in 31 consecutive hyperlipidemic patients treated with 4–6 g/day for 6 weeks [58]. A similar activity was reported with α-tocopheryl nicotinate [59], whereas a lesser activity effect (−21%) was reported with niceritrol, an ester containing four nicotinic acid (NA) residues, and which was somewhat better tolerated [60].

In order to assess the mechanisms beneath Lp(a) reduction, Seed et al. [61] tested NA (1 g t.i.d.) in patients with elevated Lp(a) levels. An impressive 36.4% Lp(a) reduction was found without any impact on Lp(a) catabolism. This indicated the major activity of NA on Lp(a) synthesis (Table 1). The authors also tested acipimox, a more powerful anti-lipolytic agent with better tolerability, but found no activity on Lp(a).

In more recent years, the combination of NA with laropiprant, a selective antagonist of the PGD2-receptor subtype-1, was extensively investigated. A sub-analysis of the HPS2-THRIVE study showed that, at 1 year, allocation to niacin-laropiprant resulted in an overall Lp(a) mean reduction of 12.2 nmol/L, which became 33.8 nmol/L in the group with Lp(a) baseline levels ≥ 128 nmol/L. Overall, the percentage reduction was 31 (95% CI 28–33%), attenuating to 36% and 18% across quintiles by increasing baseline [62]. Overall, patients who may benefit the most were those with small isoform sizes and highest baseline Lp(a) levels. The Lp(a) reduction with this combination was confirmed in patients with an established Lp(a) genotype (OMIM 152200) [63]. While the data showed an inverse correlation between baseline Lp(a) concentrations and *LPA* genotype, there was no clear difference in the Lp(a)-lowering activity of the NA–laropiprant association (mean around 25%) in the different genotypes.

Recently, the addition of PCSK9 inhibitors to a background of niacin has been associated with a roughly 15% reduction in Lp(a) beyond that achieved with niacin monotherapy [65].

## 4. PCSK9 Inhibitors

The growing use of PCSK9 inhibitors has raised the question whether they reduce Lp(a) to such an extent that enables the CV benefits linked to Lp(a)-lowering [66,67]. Although PCSK9 inhibitors overall reduce Lp(a) by a mean of 20% (Table 2), the mechanisms beneath Lp(a) reduction are uncertain [68]. They range from a key role of the LDLR in Lp(a) catabolism to a reduced Lp(a) synthesis. Recently, a further mechanism has been hypothesized, i.e., Very-Low-Density Lipoprotein (VLDL)–apoE production could influence Lp(a) production and/or assembly [69]. Epidemiological studies have shown differences in plasma Lp(a) concentrations between apoE genotypes based on the three major E isoforms (ε2/ε3/ε4) [70]. Finally, although not strictly related to PCSK9 inhibition, an important contribution to Lp(a) release has come from Ma et al., reporting that the production rate of apo(a) is raised in subjects with elevated Lp(a), compared to those with normal levels, with no differences in apo(a) fractional catabolic rate between high and low Lp(a) groups [53]. Thus, elevated plasma Lp(a) concentration appears to be a consequence of the increased hepatic production of Lp(a) particles [53].

Relative to the clinical data, evolocumab results in significant dose-related reductions in Lp(a). With this agent, the mean percentage reduction was larger in patients with baseline Lp(a) of ≤ 125 nmol/L, whereas the absolute reduction was higher in those with > 125 nmol/L [71]. Lp(a) reductions may be achieved by two different mechanisms: when given as monotherapy, evolocumab reduces the production rate of Lp(a), not the fractional catabolic rate (FCR) [72]; when administered in combination with atorvastatin, the FCR of Lp(a) rises significantly, without alterations of the production rate. Concerning alirocumab, a reduction of plasma Lp(a) levels (−18.7%) has been reported and associated with the trend of an increased median FCR for Lp(a), and no change in the production rate [73]. The same conclusions have been reached by Watts et al. [74], reporting that in ASCVD patients with elevated Lp(a) [mean Lp(a) mass 180 mg/dL] and on statins, alirocumab lowers plasma apo(a) pool size and raises the FCR of apo(a) without affecting apo(a) production rate.

The impact of PCSK9 antagonism on Lp(a) levels has also been confirmed by genetic analysis. Among carriers of Y142X (*rs67608943*) and C679X (*rs28362286*) of the REGARDS study, median Lp(a) levels were 63.2 nmol/L in carriers, compared to 80.4 nmol/L in non-carriers, respectively [75]. One hypothesis linking PCSK9 and Lp(a)-lowering may rely on the dramatic LDL-C reduction driven by PCSK9 inhibition. This could reduce a possible competitor for the binding to the LDLR, although targeted studies did not clearly indicate an involvement of the LDLR in Lp(a) uptake [76]. Although not supported by in vitro data, it might also be possible to hypothesize a direct binding between PCSK9 and Lp(a) [77,78]. Interestingly, after treatment with an anti-Lp(a) antibody, total PCSK9 levels did not change, whereas there was a steady decrement in PCSK9 bound to Lp(a) [PCSK9-Lp(a)] [79]. However, since only 1 out of 175 Lp(a) particles binds to PCSK9, this would represent a very small percentage of total Lp(a) that is complexed [80]. Moreover, a study by our group showed that, irrespective of the presence of Lp(a), the analysis of PCSK9 lipoprotein distribution by Fast Protein Liquid Chromatography (FPLC) is not affected [81].

In a pre-specified analysis of the ODISSEY OUTCOMES Trial, Lp(a) levels were monitored at randomization and at 4 and 12 months [82]. The authors evaluated total Lp(a) changes vs. LDL-C corrected, and also non-LDL-C corrected at baseline. This approach allowed the identification of the real effect on Lp(a) reduction. Median relative and absolute changes from baseline to month 4 were −23% and −5.0 mg/dL, respectively. In patients with Lp(a) baseline < 6.7 mg/dL (quartile 1; Q1), essentially no changes in Lp(a) were found, whereas at month 4, 80% of the patients in the other quartiles showed reductions. In the upper quartile (≥ 59.6 mg/dL), reductions were most significant (−20.2 mg/dL) and correlated with changes in LDL-C, both corrected and non-corrected. The reduction of Lp(a), also when corrected for LDL-C, independently predicted a lower risk of major adverse cardiovascular events (MACE). Lp(a) strata were < 6.7 mg/dL (Q1), from 6.7 mg/dL to 21.2 mg/dL (Q2), from 21.2 mg/dL to < 59.6 mg/dL (Q3), and ≥ 59.6 mg/dL (Q4). According to this distribution, the HR relative to MACE and the number-needed-to-treat (NNT) were 0.95 (0.79–1.15) and 238 for Q1; 0.85 (0.71–1.03) and 69 for Q2; 0.79 (0.66–0.94) and 43 for Q3; 0.83 (0.70–0.98) and 49 for Q4 [82]. Overall, a 1-mg/dL reduction of Lp(a) with alirocumab was associated with an HR of 0.994 (95% CI, 0.990–0.999%), thus allowing us to conclude that the risk-reduction associated with Lp(a) lowering was of the same order of magnitude as LDL-C on a per mg/dL basis [83]. The ODISSEY OUTCOMES trial also reported that in patients with acute coronary syndrome, alirocumab reduced the occurrence of peripheral artery diseases according to the magnitude of Lp(a) lowering. One mg/dL change in Lp(a) levels corresponded to a HR for peripheral artery disease or venous thromboembolism of 0.991 (0.982–1.000; *p*= 0.04). 

Overall, the same conclusions were reached in the FOURIER trial [84]. Evolocumab given for 48 weeks significantly reduced Lp(a) by a median of 26.9%. This percentage was correlated to that of LDL-C at the same interval (r = 0.37; 95% CI 0.36–0.39%). The risk of coronary death, myocardial infarction (MI) and coronary revascularization were reduced by 23% in patients with baseline Lp(a) > median (37 nmol/L equal to 13 mg/dL), while in individuals with baseline Lp(a) < median the risk reduction was only 7% (Table 2). When a threshold of 50 mg/dL was set, the NNT was 41 for the patients above the threshold and 71 for those below. The risk of MACE was the lowest for patients achieving both Lp(a) and LDL-C targets, i.e., ≤ 50 and 70 mg/dL, respectively, compared to patients who did not achieve those targets [84]. Interestingly, this evidence is similar to one observed in a pooled analysis from phase 3 ODYSSEY trials. Alirocumab was superior to placebo in reducing Lp(a) by 20–25%, an effect leading to a 12% CV-relative risk-reduction. When patients were stratified according to Lp(a) levels, those grouped as ≥ 50 mg/dL had a relative risk reduction of 40%, compared to 6% occurring in those ones achieving Lp(a) < 50 mg/dL [85]. Finally, a further sub-analysis of the FOURIER trial reported that the higher the Lp(a) levels are, the greater the risk of a subsequent aortic stenosis or aortic valve replacement [86]. Overall, this observation complements that of Langsted et al., reporting that loss-of-function PCSK9 R46L carriers had lower levels of Lp(a) and a reduced risk of aortic valve stenosis compared to non-carriers [87]. FOURIER and ODYSSEY studies confirmed that Lp(a)-lowering can be an independent contributor to MACE reduction in ACS patients [82], a concept reinforced by the conclusions of the FOURIER study, in which higher levels of Lp(a) were associated with a raised risk of CVD events independent of LDL-C [84].

In patients with Lp(a) basal levels of 80 mg/dL, evolocumab did not reduce arterial wall inflammation in the presence of modest falls in Lp(a) (−14%). The arterial benefit of LDL-C reduction may be blunted in patients with persistently elevated Lp(a). This observation may open a new dawn in CV prevention since, so far, in FH patients, changes in arterial wall inflammation have been significantly correlated with LDL-C change, whereas no correlation has been demonstrated for Lp(a) [88].

A new antagonist of PCSK9 with a different mode of action, inclisiran, acting as a silencer RNA (siRNA) for the PCSK9 gene, has also been evaluated in terms of Lp(a) reduction. Patients were given single (200, 300 and 500 mg) or two dose starting regimens (100, 200 or 300 mg on days 1 and 90). In addition to the reduction of LDL-C and apo B levels, 80% of the participants showed a reduction of Lp(a) levels at the end of the trial. However, due to the very large variability of Lp(a) concentrations, none of the differences were significant [89] (Table 2). Recently, in ASCVD patients (ORION-10 trial) or in patients with an equivalent ASCVD risk (ORION-11 trial), inclisiran (300 mg) was superior to the placebo in reducing Lp(a) levels by 25.6% and 18.6%, respectively (both placebo adjusted) [90]. In adult HeFH patients with LDL-C of 153 mg/dL (3.95 mmol/L), administration of inclisiran (300 mg) lowered LDL-C by 47.9% (vs. placebo), and Lp(a) by 17.2% (vs. baseline) [91].

## 5. Other Approaches to Lower Lp(a) Levels

*Fibrates.* An impressive reduction of Lp(a) after bezafibrate was reported [92], i.e., up to 39% in patients with Lp(a) > 30 mg/dL. A somewhat lower reduction (−13%) was earlier reported by Maggi et al. [93], and a similarly modest activity was described for gemfibrozil [94]. These and other studies have led to the conclusion that fibrates are not the drugs of choice for managing Lp(a) elevations [95], although a meta-analysis comparing fibrates and statins indicated that the former ones do not lead to Lp(a) rises, as occurs with statins [96].

*Vitamin C.* A fascinating correlation between elevated Lp(a), low vitamin C and atherosclerosis was earlier described by Rath and Pauling [97]. These authors reported the accumulation of Lp(a) in atherosclerotic lesions of the hypoascorbemic guinea pig, and hypothesized that Lp(a) may be a surrogate for ascorbate in species unable to synthesize it. The presence of Lp(a) could provide properties shared with ascorbate, such as improved wound-healing and prevention of lipid peroxidation [31]. This hypothesis was not confirmed by other authors, and did not find support in the clinic, where ascorbic acid supplementation (1 g/day) did not significantly affect Lp(a) concentrations [98]. 

*Sex hormone therapies*. Post-menopausal norethisterone treatment led to a 47% reduction of Lp(a) levels [99], whereas the effect was found to be less marked in post-menopausal women given low doses of estradiol valerate (2 mg/day) [100]. Larger effects (−17% to −23%) were instead reported in post-menopausal women on conjugated estrogens with or without medroxyprogesterone acetate [101]. However, hormone replacement therapy cannot be recommended for the sole purpose of lowering Lp(a) [102]. A reduction of 40% in Lp(a) levels was shown after tamoxifen administration in patients diagnosed with breast cancer [103]. Finally, dramatic Lp(a) reductions have been reported in body builders on stanazolol [−65% Lp(a)] [104] or danazol (−78%) [105]. 

## 6. Lipoprotein Apheresis (LA) to Reduce Lipoprotein(a)

Although LA, within the therapeutic regimen of lipid disorders, is often considered as a therapy of last resort, guidelines differ in defining which patients to treat, and under which circumstances [106]. Some of these guidelines recommend apheresis as a first-line treatment in patients with HoFH, and after drug therapy failure in patients with heterozygous (He)FH, with differences also in LA treatment frequency (weekly or biweekly) [107,108]. LA is highly effective in reducing Lp(a) levels, i.e., approximately 57 mg/dL or 39% when comparing Lp(a) levels measured before the start of apheresis and the interval mean values during the apheretic procedure [24]. Currently, LA is mainly used in two different clinical settings, i.e., significantly elevated LDL-C or Lp(a). In patients with hyperlipoproteinemia(a), and on maximally tolerated lipid-lowering medications, LA seems to lower the progression of atherosclerosis, leading to a reduced number of CV events [109,110,111]. In the case of CV events, when comparing the time intervals from the start of LA to a similar time on no LA, LA may lead to a more than 80% risk-reduction [112,113,114]. Evidence from case studies showed that apheresis is more effective in patients with elevated Lp(a) levels when compared with those with normal concentrations [115]. Data from the Low-Density Lipoprotein Apheresis Coronary Atherosclerosis Prospective Study (L-CAPS) trial showed that intensive cholesterol lowering by apheresis prevented coronary atherosclerosis progression. The restenosis rate was 12.5% in the FH patients whose Lp(a) levels dropped more than 50%, compared to 53% in those with lesser Lp(a) reductions [116].

The European Atherosclerosis Society Consensus Panel recommended that Lp(a) levels should be reduced below 50 mg/dL in extreme cases by LA [26]. The HEART UK Guidelines recommended LA for patients on maximally tolerated lipid-lowering therapies and with progressive coronary heart disease and persistent elevations of Lp(a) > 60 mg/dL and LDL-C > 125 mg/dL (3.23 mmol/L) [117]. In Germany, Lp(a) levels exceeding 60 mg/dL, along with progressive CVD, were approved as an indication for regular LA in 2008 by the Joint Federal Committee. The American Society for Apheresis recommends the use of LA for the treatment of elevated Lp(a) (> 50 mg/dL) in CVD patients [118]. In the US, the Food and Drug Administration approved LA for HoFH patients with LDL-C > 500 mg/dL (12.92 mmol/L) (beginning in childhood), for HeFH with LDL-C >300 mg/dL (7.75 mmol/L) and no sign of CVD, or with known CVD and LDL-C > 200 mg/dL (5.17 mmol/L) [119]. In Japan, LA is approved for patients with CVD and total cholesterolemia > 250 mg/dL (6.46 mmol/L) [120]. As elsewhere reviewed in detail [121], available LA techniques are categorized as selective (immune adsorption, dextran sulfate adsorption, heparin precipitation, cascade filtration and polyacrylamide adsorption) or non-selective (plasma exchange; Table 3). 

Briefly, the most commonly used LA systems share the specific adsorption of apoB, constitutive of VLDL, LDL and Lp(a). ApoB-containing lipoproteins are removed either by precipitation in the excess of heparin (HELP Braun) at acidic pH (5.2), or by the binding of positively charged apoB to the negatively charged surface dextran sulfate coupled to cellulose beads (DSA), or by apoB-immunoabsorption [123]. While removal efficiency differs among the systems [124], in FH patients all techniques rapidly remove LDL-C (55–70%) as well as Lp(a) mass (50–60%) [125]. Later publications by the same group indicated a higher removal rate [24]. However, the percentage changes reported in Table 3 reflect not only the intrinsic efficiency of each method in removing plasma lipoproteins, but also differences in the volume of blood or plasma treated and the extent of haemodilution caused by the anticoagulant used [126]. Typically, from 4- to 6-L exchanges of plasma are carried out for 2–4 h weekly or biweekly [127].

An inherent drawback of LA is the cyclical rebound of LDL-C within 1 to 2 weeks between apheretic procedures. Lp(a) rebounds at a slower rate than LDL-C, but with a similar monoexponential function [106]. Thus, despite an acute decrement of 70–75%, regular apheresis can translate into a significant interval mean Lp(a) reduction between 25% and 40% [128]. Specifically, depending on the Lp(a) baseline and the selected interval, a biweekly apheresis generally results in a much lower interval mean reduction (20%), compared to a weekly procedure (36%) [109,129]. This bulk of reduction also persisted in patients undergoing long-term LA, wherein mean pre-apheresis levels of Lp(a) were reduced by 22% after 1 year, and by 19% after three years [130,131]. To reduce the lipoprotein rebound, one strategy is the use of lipid-lowering therapies in between the procedures. The effect of PCSK9 inhibition on the frequency of standard LA treatments was the focus of the ODYSSEY ESCAPE study [132]. Although LA was discontinued in 63.4% of patients on alirocumab and the rate was reduced at least 50% in 92.7% of patients, any additive effect of alirocumab on top of LA on Lp(a) levels was not found [133]. Santos et al. [134], in HoFH and severe HeFH patients, showed that long-term administration of evolocumab (over a median of 4.1 years) allowed 3 out of 34 HoFH, and 13 out of 27 severe HeFH, patients to discontinue LA. The DE LAVAL study showed that among patients on weekly or every-two-week LA and with a moderate- to high-intensity statin background, evolocumab led > 50% of patients to reach LDL-C < 68 mg/dL (1.76 mmol/L), demonstrating that evolocumab may, in these cases, replace LA [135]. Finally, the EVOLAFER01 trial enrolling HeFH patients on long-term LA therapy reported evolocumab to be superior to LA in reducing LDL-C and Lp(a), and that the combination of evolocumab plus LA could represent a therapeutic alternative to lowering LDL-C and Lp(a) levels in patients with very high CV risk [136]. However, these studies did not specifically address the lowering of Lp(a). Relative to real-life studies on long-term apheresis patients, the use of PCSK9 inhibitors seems unable to replace LA in 75% of patients, and in 45% of people with isolated hypercholesterolemia, thus pointing out the role of LA as a last resort lipid-lowering option [137].

From a clinical point of view, LA can also modify a number of pathological processes associated with CVD: it improves markers of vascular inflammation, and decreases fibrinogen, E-selectin, vascular cellular adhesion molecule-1, intercellular adhesion molecule-1, monocyte chemoattractant protein-1, lipopolysaccharide binding protein, matrix metalloproteinase and tissue inhibitor of metalloproteinase [138,139,140,141]. Among these effects, the most important one is the restoration of an effective myocardial blood flow induced by LA, well established many years ago [142] and recently confirmed [143], in clinical cases of refractory angina associated with raised Lp(a). Improvement of myocardial blood flow can be assessed, among other means, by Positron Emission Tomography (PET) and by echo-doppler sonography [144]. These hemodynamic effects are not far from those exerted by percutaneous transluminal coronary angioplasty (PTCA). This additional value attributable to Lp(a) apheresis in this condition makes this technique more impactful in cardiological practice. Furthermore, cardiac magnetic resonance imaging allows one to detect treatment-related changes in regional myocardial perfusion in patients with elevated Lp(a) (≥ 117 mg/dL) and coronary artery disease undergoing LA [145]. The possible application of selective Lp(a) apheresis also appears to offer a promising approach to the prevention of Lp(a) associated to CV risk. The specific immunosorbent column named ‘Lp(a) Lipopak’ (POCARD, Moscow, Russia) represents, so far, the most efficient LA able to selectively reduce Lp(a) levels by 88%, compared to pretreatment [146] (Table 3). In patients with ischemic heart disease, an 18-month application of Lp(a) apheresis reduced the diameter of stenosis (−5.05%) and total atheroma volume (−4.60 mm^3^), and raised minimal coronary lumen diameter (+14%) [147]. Another crucial aspect worth mentioning is the ability of Lp(a) to bind and transport oxidized phospholipids (OxPLs) that represent a key component of the atherothrombotic risk associated with Lp(a) [148]. OxPLs are sequestered on Lp(a) and subjected to degradation by the Lp(a)-associated lipoprotein-associated phospholipase A2 (Lp-PLA2), suggesting that Lp(a) might be a scavenger of OxPL [149]. Upon LA, there is an acute reduction of Lp-PLA(2) (roughly −20%), an effect independent of LDL-C when LA becomes a chronic treatment. This could explain the potential mechanism by which LA reduces coronary heart disease risk [140].

## 7. Antisense Antinuocleotide

In view of the apparent increase of Lp(a) synthesis in carriers of elevated Lp(a), and consequent reductions by PCSK9 antagonists, the potential of biosynthetic drug treatments appears to be best targeted to synthesis. In particular, the use of appropriate antisense oligonucleotides (ASOs) appears to be most suitable [150]. The first developed APO-(a)Rx by IONIS was a second generation 2-O-(2-metoxyethyl) (2-MOE)-modified ASO with improved potency, duration and tolerability. Designed to reduce the synthesis of apo(a) in the liver, this ASO was tested in a double-blind phase I study, at doses between 50 and 400 mg/day. Single doses did not lead to any reduction of Lp(a) at day 30, but six doses of 100–300 mg resulted in a dose-dependent percentage reduction of Lp(a) concentrations of 39.6% from baseline in the 100 mg group, up to 77.8% in the 300 mg group [151].

To assess the potential validity of this approach, also the frequently concomitant presence of stenotic aortic valves, a follow up study evaluated 64 participants with elevated Lp(a) concentrations (52–182 mg/dL) [151]. This time the variant IONIS-APO(a)Lrx was tested, i.e., a ligand-conjugated ASO with a triantennary N-acetylgaloctosamine (GalNAc) covalently attached, to allow rapid and specific uptake by hepatocytes. In a proof-of-concept phase 1/2a trial in healthy volunteers with Lp(a) levels ≥ 30 mg/dL, this ASO led to a stepwise reduction in fasting Lp(a) levels, the maximal benefit occurring at day 30: −24.8% (10 mg), −35.1% (20 mg), −48.2% (40 mg), −82.5% (80 mg) and −84.5% (120 mg) [151] (Table 4).

The most recent multicenter international dose-ranging phase 2b trial was carried out on 286 patients with pre-existing CVD and baseline Lp(a) > 60 mg/dl. Doses were 20, 40, 60 mg or placebo every 4 weeks (Q4W), 20 mg every 2 weeks (Q2W) or 20 mg once weekly (QW). Duration was 25–27 weeks. IONIS-APO(a) Lrx resulted in a dose-dependent reduction of Lp(a) in all treated groups. Mean percentage decreases were of 35% at 20 mg Q4W, 56% at 40 mg Q4W, 58% at 20 mg Q2W, 72% at 60 mg Q4W and 80% at 20 mg QW. Placebo (saline) only reduced Lp(a) by 6%. The product was well tolerated, with no changes in platelet number or liver and renal function, and no influenza-like symptoms. Injection site reactions occurred in approximately 7% of cases, the most common associated adverse reactions being erythema (26%); only one patient discontinued treatment because of an injection site reaction. There were no potential pro-inflammatory effects as assessed from the determination of high-sensitivity CRP levels, which were maintained between 2 and 3 mg/L across groups with no statistically significant differences [152] (Table 4).

Further, looking at the benefit driven by a dramatic Lp(a) lowering, in CVD patients with elevated Lp(a), IONIS-APO(a) Lrx led to a reduction of multiple immune response-related pathways, including interferon (IFN)α, IFNγ and Toll-like receptor (TLR) pathways. Expression of C-C chemokine receptor type 2 (CCR2), CX3C chemokine receptor 1 (CX3CR1) and TLR2 were also reduced. Trans-endothelial migration activity of monocytes was reduced (−22%). Conversely, a modest Lp(a) lowering driven by PCSK9 inhibition (evolocumab) did not result in such effects, despite a 65% reduction in LDL-C [153].

## 8. Future Perspectives and Conclusions

In a scenario missing a pharmacological approach to lowering Lp(a) to the extent required to achieve a CV benefit, in patients with progressive ASCVD and high plasma Lp(a), a potentially valuable therapeutic option is LA, alone or in combination with PCSK9 inhibitors. However, because of the difficult standardization of apheretic modalities, there is still an unclear response to the main question of whether the Lp(a) reduction will have beneficial effects on the CV system, even in the clinical setting of isolated elevated Lp(a) levels. The prospective study by Roeseler et al. [4] confirmed a significant CV benefit over 5 years of follow-up with different apheretic techniques in patients with hyperlipoproteinemia(a), with a significant reduction of CV events over the prior non-apheretic treatment. However, it should be taken into consideration that these studies all suffer from potential confounding, such as the lack of any scope for ruling out the effect of LA on other drivers of events, such as LDL or fibrinogen. Moreover, to determine the contribution of Lp(a) apheresis, a study with a controlled prospective randomized design, enrolling only patients with elevated Lp(a), should be planned [128].

While the use of PCSK9 inhibitors has led to a mean fairly modest reduction in Lp(a) levels (20–25%), there is no currently accepted indication for the use of these biosynthetic compounds to treat hyperlipoproteinemia(a) [154]. However, in combination with LA, the use of PCSK9 inhibitors decreases the need for apheresis, as most patients with HeHF and other forms of hypercholesterolemia respond very well to this therapy. Instead, contrasting conclusions have been reached in the case of statins, while ezetimibe monotherapy provided a modest 7% reduction [155]. Relative to mipomersen, although the potential benefit in reducing Lp(a) was between 20% and 50% [156], the manufacturing of the compound was discontinued (2018) and the product is not clinically available [150]. Interestingly, although not approved for clinical use [157], inhibitors of cholesteryl ester transfer protein lowered Lp(a) in a range between 25% and 40% [156]. Finally, in the era of RNA-based therapies, a new dawn is represented by APO(a)L_rx_ [152]. The Lp(a) Horizon trial will test the effect of the ASO TQJ230 against apo(a) in patients with previous MI, stroke or symptomatic peripheral artery disease, with an optimized LDL-C lowering therapy and Lp(a) ≥ 70 mg/dL. The estimated completion date is March 2024 (NCT04023552). However, future safety and cost-effectiveness studies are required to establish the role of these new agents in clinical practice.

Finally, moving to a biochemical point of view, adjusting LDL-C concentration for Lp(a)-C improves the diagnostic accuracy of the Dutch Lipid Clinic Network and Simon Broome criteria, especially for patients with Lp(a) > 100 mg/dL and LDL-C < 251 mg/dL (6.49 mmol/L) [36]. It should be taken into consideration that each Lp(a) particle is composed of about 30−45% of cholesterol, i.e., Lp(a)-C is equal to Lp(a) total mass in mg/dL/3. Thus, when Lp(a) total mass is high, the Lp(a)-C contribution to the LDL-C is significant [158]. Considering that *Lp(a)-corrected LDL-C (mg/dL) = LDL-C (mg/dL)*—*[Lp(a) (mg/dL) × 0.30]*, Lp(a)-corrected LDL-C is 55−70 mg/dL in a person with an LDL-C concentration of 100 mg/dL and an Lp(a) concentration of 100 mg/dL [159,160]. If LDL-C is expressed in mmol/L the formula is as follows: *Lp(a)-corrected LDL-C (mmol/L) = LDL-C (mmol/L)—[Lp(a) (mg/dL) × 0.0078]* [161]. Although the confounding effect of Lp(a) cholesterol on the phenotypic diagnosis of FH in people with hyperlipoproteinemia(a) has been highlighted, to confirm its practical implications for the care of patients requires further studies [74].

## Figures and Tables

**Table 1:** Impact of synthetic lipid-lowering drugs on lipoprotein(a) levels.

Drug	Study	Lp(a) ^1^ Changes
Statin	5256 patients (1271 on placebo and 3885 on different statins) from six randomized trials [52]	from +11.6% to +20.4% (pravastatin group) from +18.7% to +24.2% (atorvastatin group) mean absolute increase was 18.1 mg/dL in the group with Lp(a) ≥ 30–50 mg/dL mean absolute increase was 10.3 mg/dL in the group with Lp(a) ≥ 50 mg/dL
	JUPITER^2^(rosuvastatin 20 mg/d) [57]	The median change in Lp(a) with rosuvastatin and placebo was zero Placebo arm: median (25th and 75th) was 23 nmol/L (10–48) Rosuvastatin arm: median (25th and 75th) was 24 nmol/L (10–51)
Nicotinic acid	Hyperlipidemic patients [58]	−38% (95% CI 28–47%)
	Hyperlipidemic patients with Lp(a) concentrations ⩾ 18 mg/dL [59]	from −27.0 ± 5.4 to −20.6 ± 4.1
	Normolipidemic patients with coronary artery disease [60]	−21%
	Patients with type II hyperlipidaemia and plasma Lp(a) concentration ≥ 30 mg/dL [61]	−36.4%
	HPS2-THRIVE^3^ [62]	Lp(a) mean reduction of 12.2 nmol/L, which became 33.8 nmol/L in the group with Lp(a) baseline levels ≥ 128 nmol/L
	Patients with different baseline Lp(a) concentrations [63]	−19.2% ± 3.7% if Lp(a) was < 50 mg/dL −20.7% ± 5.4% if Lp(a) was between 50 and 120 mg/dL −29.5% ± 2.2% if Lp(a was >120 mg/dL)

^1 ^Lp(a), lipoprotein(a); ^2^ JUPITER, Justification for the Use of Statins In Primary Prevention; ^3^ HPS2-THRIVE, Treatment of HDL to Reduce the Incidence of Vascular Events HPS2-THRIVE.

**Table 2:** Impact of PCSK9 ^1^ inhibitors on lipoprotein(a) levels.

Drug	Study	Lp(a) ^2^ Changes
PCSK9 mAbs ^4^	Pooled analysis from four phase II studies with evolocumab [71]	*Baseline Lp(a) levels ≤ 125 nmol/L* −16.1% (70 mg Q2W)^3^ −27.6% (105 mg Q2W) −33.2% (140 mg Q2W) −21.0% (280 mg Q4W) ^3^ −25.3% (350 mg Q4W) −28.7% (420 mg Q4W) *Baseline Lp(a) levels > 125 nmol/L* −7.5% (70 mg Q2W) −17.4% (105 mg Q2W) −20.0% (140 mg Q2W) −11.8% (280 mg Q4W) −11.1% (350 mg Q4W) −16.1% (420 mg Q4W)
	Healthy patients (alirocumab) [73]	−18.7% (from −30.6% to −11.2%)
	ODISSEY OUTCOMES trial (alirocumab) [82]	*Baseline Lp(a) levels = 21.2 mg/dL (median)* −5.0 mg/dL (overall) −1.6 mg/dL (Q1, < 6.7 mg/dL) ^5^ −4.8 mg/dL (Q2, 6.7 to < 21.2 mg/dL) −13.4 mg/dL (Q3, 21.2 to < 59.6 mg/dL) −20.2 mg/dL (Q4, > 59.6 mg/dL)
	FOURIER trial (evolocumab) [84]	*Baseline Lp(a) 37 nmol/L (from 13 nmol/L to 165 nmol/L; median)* −26.9% (from −6.2% to −46.7%)
PCSK9 antisense	ORION-1 (inclisiran) [89]	from −14% to −18% (single dose group) from −15% to −26% (double dose group)
	ORION-9 (inclisiran)	Lp(a): −17.2% vs. baseline
	ORION-10 and ORION 11 (inclisiran) [90]	*ORION 10* Lp(a): −25.6% (placebo adjusted) *ORION 11* Lp(a): −18.6% (placebo adjusted)

^1^ PCSK9, proprotein convertase subtilisin/kexin type 9; ^2^ Lp(a), lipoprotein(a); ^3^ QW, once weekly; Q2W, every two weeks; QW4, every 4 weeks; ^4^ mAb, monoclonal antibodies; ^5^ Q, quartile.

**Table 3:** Impact of different lipoprotein apheretic approaches on LDL-C and Lp(a) levels.

Lipoprotein Apheresis	Description	Reduction
Adsorption	DALI (*direct adsorption of lipoproteins*). Electrostatic interaction of negatively charged polyacrylate anions with positively charged apoB	LDL-C ^1^: 53–76% Lp(a) ^2^: 28–74%
	DSA (*Dextran sulfate-cellulose-based-adsorption*). Electrostatic interaction of negatively dextransulfate with positively charged apoB	LDL-C: 49–75% Lp(a): 19–70%
	IMA (*immunoadsorption*). Plasma is passed through columns containing polyclonal anti-apoB100 antibodies	LDL-C: 62–69% Lp(a): 51–71%
	Lipopac (*Lp(a) specific*). Plasma is passed through columns containing polyclonal anti-apo(a) antibodies	LDL-C: 7% Lp(a): 59–88%
Filtration	MONET (*Membrane Filtration Optimized Novel Extracorporeal Treatment*). Series of filters eliminate LDL and Lp(a) from plasma based on size properties	LDL-C: 52–62% Lp(a): 53–59%
	Lipid filtration. Series of filters eliminate LDL and Lp(a) from plasma based on size properties	LDL-C: 61% Lp(a): 61%
Precipitation	HELP (*Heparin-induced extracorporeal LDL precipitation*). Precipitation of a complex consisting of heparin, LDL, Lp(a), and fibrinogen at pH = 5.2	LDL-C: 55–61% Lp(a): 55–68%
Plasma Exchange	Although plasma exchange is still used in some centers, it is increasingly being replaced by selective LA, except when treating patients with severe hypertriglyceridemia [122]	

Variability among procedures relates partially to differences in the volume of plasma and blood treated. ^1^ LDL-C, low-density lipoprotein-cholesterol; ^2^ Lp(a), lipoprotein(a).

**Table 4:** Impact of antisense oligonucleotide APO(a) on lipoprotein(a) levels.

Drug	Study	Lp(a) ^1^ Changes
Antisense antinucleotide	APO(a)Rx [151]	Lp(a) 125–437 nmol/L: −66.8% Lp(a) concentration ≥ 438 nmol/L: −71.6
	APO(a)L_Rx_ [151]	*Single-ascending-dose group:* −24.8% (95% CI 3.1–67.1) at day 30 (10 mg group) −35.1% (2.2–78.8) at day 30 (20 mg group) −48.2% (10.9–78.4) at day 30 (40 mg group) −82.5% (50.5–109.2) at day 30 (80 mg group) −84.5% (65.2–112.6) at day 30 (120 mg group) *Multiple-ascending dose group:* −59.4% (95% CI 33.5–79.1) at day 36 (10 mg group) −72.3% (51.6–87.7) at day 36 (20 mg group) −82.4% (67.6–99.8) at day 36 (40 mg group)
	APO(a)L_Rx_ [152]	*Lp(a) > 60 mg/dl (150 nmol/L)* −35% (20 mg of AKCEA-APO(a)L_Rx_ Q4W) −56% (40 mg of AKCEA-APO(a)L_Rx_ Q4W) −58% (20 mg of AKCEA-APO(a)L_Rx_ Q2W) −72% (60 mg of AKCEA-APO(a)L_Rx_ Q4W) −80% (20 mg of AKCEA-APO(a)L_rx_ QW)

^1^ Lp(a), lipoprotein(a).
